# Research on Multi-Core Curvature Sensing Measurement Based on PPP-BOTDA

**DOI:** 10.3390/s24186023

**Published:** 2024-09-18

**Authors:** Zijuan Liu, Yongqian Li, Lixin Zhang, Lei Wang

**Affiliations:** 1 Department of Electronic and Communication Engineering, North China Electric Power University, Baoding 071003, China; liyq@ncepu.edu.cn (Y.L.); lxzhang@ncepu.edu.cn (L.Z.); 2 Hebei Key Laboratory of Power Internet of Things Technology, North China Electric Power University, Baoding 071003, China; 3 School of Energy Storage Science and Engineering, North China University of Technology, Beijing 100144, China; 1172101044@ncepu.edu.cn

**Keywords:** pre-pumped, double peak, two-sided band interference, bending measurements, curvature

## Abstract

To address the issue of spatial resolution limitations in traditional Brillouin optical time-domain analysis systems due to phonon lifetime constraints, we employed pre-pumped pulse technology. Additionally, to mitigate the double-peak phenomenon observed in pre-pumped Brillouin optical time-domain analysis systems, we implemented a two-sided band interference method to reduce the linewidth of the double-peak fitting. We conducted bending measurements on three eccentric cores and intermediate cores spaced 120° apart. Our results demonstrate that the system described in this paper can achieve a spatial resolution of 30 cm, with bimodal linewidths of 23.1 MHz and 16.0 MHz. Using the parallel transmission frame algorithm, we determined the curvature of a seven-core fiber with a curvature diameter of approximately 10 cm to be 20.67 m^−1^, with an error margin of 3.2%.

## 1. Introduction

Fiberoptic shape-sensing technology, an emerging field in recent years, has advanced from traditional bending measurement and has gained widespread attention across various domains. Applications include software operators in the medical field, deformation monitoring of aircraft skins in the aerospace sector, online deformation monitoring of bridges, and the shape sensing of flexible robotic tentacles [1]. Accurate measurement and acquisition of the curvature of the deformation segment are crucial for the shape reconstruction of optical fibers in shape-sensing technology.

Optical fiber sensing technology offers a promising approach for curvature sensing due to its strong anti-interference capability, long measurement range, high transmission reliability and accuracy, and simple wiring [1,2,3,4]. Recent advancements in this field have focused on grating-type and interferometer-type optical fiber curvature sensors. Traditional optical fiber sensing systems typically use single-mode fiber as the sensing medium, with the fiber core located along the central axis of fiber bending, rendering single-mode fiber insensitive to bending strain [4,5]. To accurately measure the curvature of the deformation section, it is essential to utilize a core that is asymmetrically distributed relative to the central axis of fiber bending. In 2013, Roesthuis et al. embedded three single-mode fibers with fiber Bragg grating (FBG) into grooves at 120° intervals, securing them with an adhesive around a medical biopsy needle. These grooves formed curvature sensors to measure the axial strain of the needle, thus calculating its curvature [6]. In 2019, Oleg et al. designed a large-diameter four-core fiber shape sensor based on FBG with high bending sensitivity. The four cores were symmetrically arranged at 90° angles, and the sensor had a diameter of 2.1 mm, achieving a bending sensitivity of 3.6 × 10^−3^ m^−1^ [7]. In 2021, Liu Z et al. introduced a novel temperature-insensitive optical curvature sensor by writing a short-length FBG into a strongly coupled multi-core fiber (SCMCF) fused with a conventional single-mode fiber. The experimental results showed that the sensor’s sensitivity reached 15.9 dB/m^−1^ in the lower curvature range [8]. In 2022, Leal-Junior A et al. used femtosecond laser direct writing to inscribe core-skewed FBG in a transparent optical polymer (CYTOP) fiber. The results indicated that, compared to writing a center uniform fiber grating in CYTOP fiber, the wavelength drift of the skewed grating changed linearly with temperature increases while the optical power remained nearly unchanged. This sensor did not consider the cross-influence of temperature when measuring bending angles [9].

Compared to grating-type curvature sensors, those based on interference structures exhibit higher sensing sensitivity. In 2018, Wang Q et al. used multi-mode fiber fused between single-mode fiber and seven-core fiber to enhance optical coupling efficiency, employing the seven-core fiber as the primary structure for measuring curvature. This configuration achieved a maximum curvature sensitivity of 41.46 nm/m^−1^ within a bending curvature range of 0.094 to 0.567 m^−1^ [10]. In 2020, Arrizabalaga O et al. proposed a vector bending and directional discriminating curvature sensor based on asymmetric strongly coupled multi-core fiber. This sensor utilized a strongly coupled seven-core fiber and single-mode fibers fused at both ends. By optimizing the mode coupling effect of the seven-core fiber, the sensor achieved a vector bending direction sensitivity of −1.4 nm/° and a curvature sensitivity of −17.5 nm/m^−1^ [11]. In 2022, Zhang Shaoxian et al. developed a sensor by fusing single-mode fibers at both ends of a three-core fiber, with the symmetrical core capable of distinguishing bending directions. When bent towards 0°, the spectrum exhibited a blue shift with a curvature sensitivity of −29 nm/m^−1^; when bent towards 180°, the spectrum showed a redshift with a maximum curvature sensitivity of 41.09 nm/m^−1^ [12]. Grating-type optical fiber curvature sensors face issues of temperature cross-sensitivity during curvature measurement, making them unsuitable for environments with significant temperature fluctuations. On the other hand, interferometer-based optical fiber curvature sensors often have complex structures. They may require taper welding, thick taper welding, fiber punching, coating materials, or dislocation welding, either alone or in combination, resulting in low mechanical strength and difficulty in measuring a wide range of curvatures. While FBG-based curvature sensing technology offers high precision and straightforward data processing, it is constrained by the number of fiber gratings and the writing distance, limiting it to short-distance measurements and hindering strictly distributed curvature measurement.

Distributed optical fiber sensing systems (DFOSs) have garnered significant attention from scholars due to their excellent anti-electromagnetic interference capabilities, long measurement distances, high precision, and strong reliability [13,14]. Currently, curvature sensors based on DFOS primarily utilize multi-core fibers (MCFs). As early as 2006, the LUNA Technology Company of the United States used the Optical Frequency Domain Reflection (OFDR) system connected with fiber grating to achieve the curvature measurement and reduction of 1 cm spatial resolution on a 1.1 m long MCF and applied the technology to the minimally invasive navigation system Ion [15,16]. In 2012, the company laid MCF shape sensors with a length of 30 m on the surface of a 10 m flexible structure in two different ways, U-shaped and gyro-shaped, and obtained the surface shape of the flexible material through data processing [17]. In 2021, Zeen Chen et al. demonstrated a large curvature radius shape-sensing experiment with MCFs using OFDR. By reconstructing the error model, the measured strain resolution and sensing spatial resolution were optimized, and the two-dimensional circle shape with a curvature radius of 5 cm~100 cm was reconstructed. The average Euclidean distance between the reconstructed shape and the designed space curve is 3.4 mm [18]. Although high spatial resolution can be obtained by using the OFDR system for curvature measurement, the current studies all performed short distance curvature measurement within 100 m, and the sensing distance is limited. The time-domain analysis method provides a good approach for researchers. In 2016, Zhao Zhiyong and colleagues utilized a differential pulse pair-Brillouin optical time-domain analysis (DPP-BOTDA) system to investigate the bending characteristics of both the central core and the eccentric cores of a 1 km multi-core fiber, achieving a spatial resolution of 20 cm. Their experimental results indicated that the strain in the central core remained unaffected by fiber bending, while the eccentric core demonstrated high bending sensitivity [19]. In 2017, the team measured the Brillouin frequency shift strain coefficient of the seven-core fiber, finding it to be 0.0485568 ± 0.0002495 MHz/με for the central core and 0.0485888 ± 0.0002736 MHz/με for the eccentric cores [20]. 

To address the limitation of spatial resolution in traditional BOTDA sensing systems, which are typically on the order of meters, this paper employs pre-pumped pulse technology to enhance the spatial resolution to 30 cm. Temperature measurement experiments verify the double-peak phenomenon in the pre-pumped pulse BOTDA system, and the double-sideband interference method is used to reduce the linewidth of the double peak. This reduces the bimodal linewidth from 31.2 MHz and 29.6 MHz to 23.1 MHz and 16.0 MHz, effectively mitigating frequency aliasing in bimodal fitting. Bending measurements are conducted using a seven-core optical fiber. Three asymmetrical cores and intermediate cores are selected, with the intermediate cores used for temperature compensation. The curvature of the seven-core fiber, with a curvature diameter of approximately 10 cm, is calculated using the parallel transmission frame algorithm. The resulting curvature is 20.67 m^−1^ with an error margin of 3.2%, and the causes of this error are analyzed. 

## 2. Materials and Methods

The shape measurement of optical fiber is essentially an extension of distributed optical fiber strain measurement. By establishing the relationship between optical fiber strain and both bending curvature and direction angle, the measured strain information is converted into the curvature parameters of the fiber’s central axis. This allows for the reconstruction of the shape of the optical fiber or the object closely fitted with it. Therefore, accurate curvature measurement is crucial.

### 2.1. Pulsed Pre-Pumped BOTDA (PPP-BOTDA)

Due to the inhomogeneity of the optical fiber medium, scattered signals are generated during the transmission of light, primarily including Rayleigh scattering, Brillouin scattering, and Raman scattering [3,4]. Brillouin scattering can be categorized into two types based on the formation mechanism of the acoustic field in the optical fiber: Spontaneous Brillouin Scattering (SPBS) and Stimulated Brillouin Scattering (SBS). The SPBS effect produces backward-Stokes and anti-Stokes light in the fiber. When the incident light power exceeds the fiber threshold, energy exchange occurs between the forward-transmitted incident light and the backward-Stokes light, leading to an increase in the energy of the backward-Stokes light, a phenomenon known as the SBS effect. The BOTDA system operates based on the SBS principle in optical fibers. Pulsed light is incident at one end of the sensing fiber, while continuous light is incident at the other. The higher-frequency pulsed light acts as the pump light, and the lower-frequency continuous light serves as the probe light. By processing the backward-Stokes scattered light, the Brillouin gain spectrum of the entire fiber can be obtained. The BOTDA system offers high detection accuracy, long sensing distances, and a high signal-to-noise ratio. However, due to the limitation imposed by the phonon lifetime, the spatial resolution of the BOTDA system is restricted to the order of meters.

To overcome this limitation, reducing pulse width is commonly used to improve spatial resolution. However, excessively small pulse widths can cause significant broadening of the Brillouin spectrum, severely affecting measurement accuracy. Pulsed pre-pumping technology offers an effective method to achieve spatial resolution on the order of centimeters. Due to the relaxation effect of phonons, their generation and disappearance are not instantaneous during Brillouin interactions, requiring a buffering time of over 10 ns for stable and complete Brillouin amplification. Short pulses alone may not fully interact, but using a long, low-power pre-pump pulse before the short pulse can excite phonon production, preparing the system for the subsequent Brillouin process. This approach allows the Brillouin sensing process to proceed effectively, even with short pulses, by utilizing the acoustic field excited by the pre-pumped pulse.

**Figure 1:**
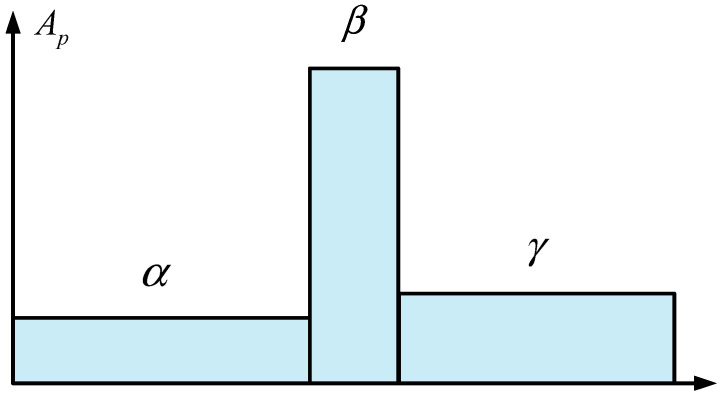
Schematic diagram of the pre-pump pulse.

**Figure 2:**
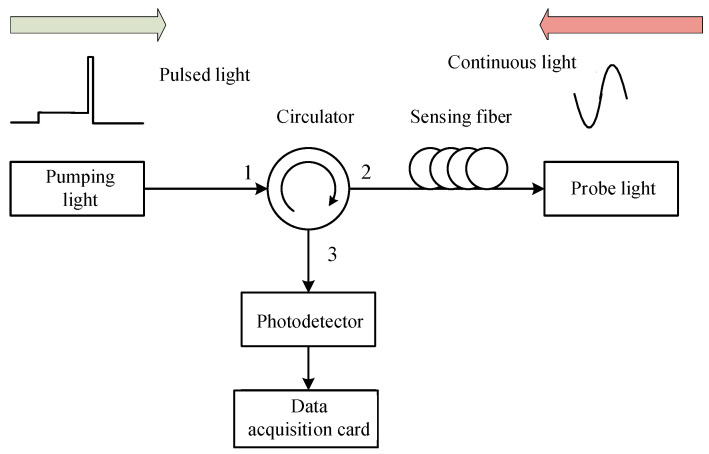
Principle of pulsed pre-pumping BOTDA fiber sensing technology.

The composition of the pulsed light is shown in Figure 1. Usually, the α and γ components are long pulses with equal phase and amplitude, and the duration is much longer than 10 ns [21,22]. The β component, located between α and γ, is a narrow pulse with a width of less than 10 ns, and its phase and amplitude are different from the other two continuous light components. The principle of BOTDA fiber sensing technology is shown in Figure 2 [23]. The step pulse consists of a high-power sensing pulse and a low-power pre-pumping pulse. First, the pre-pumped pulsed light enters the sensing fiber to fully excite the acoustic field. When the difference in the frequency of the sensing pulse light and the continuous detection light is equal to the Brillouin frequency shift in the fiber, an SBS interaction occurs between the two. By continuously adjusting the frequency of the continuous light and measuring the continuous light power coupled from the fiber, temperature and strain can be measured.

**Figure 3:**
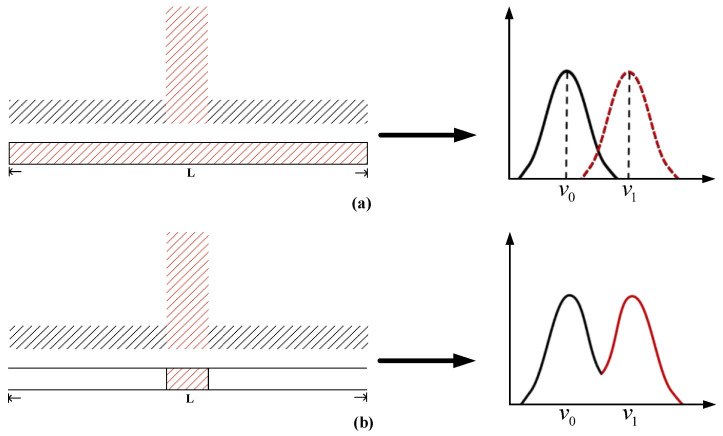
Brillouin spectrum at hot spot locations of fiber with different heating lengths in the pre-pump system: (**a**) the length of the heating section is greater than ***L***; (**b**) the length of the heating section is less than L.

In the process of BOTDA sensing by a pre-pumping pulse, the Brillouin spectrum collected at the hot spot is superimposed on the Brillouin spectrum of the entire pulse covering the fiber segment due to the non-local effect, so there will be a multi-peak structure at the hot spot. When the pre-pumped part β passes through any position along the fiber, the pre-excited steady-state phonon of part α will interact with the Brillouin of part β with a different amplitude or phase and will appear slightly changed. When detecting the Brillouin signal in the heated or stressed section of the fiber, the central Brillouin frequency across the entire fiber changes due to the effects of the heating or stress in that region. If the Brillouin frequency is not evenly distributed along the pulse length, as shown in Figure 3a (Red indicates the optical fiber at the hot spot, and black indicates the optical fiber in normal environment.), and if the optical fiber length at the hot spot (heating section/stressed section) is greater than the length covered by the pre-pumped pulse ***L***, that is, the Brillouin frequency is evenly distributed along the pulse length, then the Brillouin spectrum can be fully frequency-shifted. However, as shown in Figure 3b, when the optical fiber length at the hot spot is less than the pre-pumped pulse length ***L***, the spectrum obtained at the hot spot superimposes the Brillouin spectral component with a frequency shift at the hot spot and the spectral component without a frequency shift at other locations, resulting in non-local effects and the formation of a Brillouin peak structure [24]. The first peak is the Brillouin spectrum caused by the pre-pulse, and the second peak is the Brillouin spectrum generated by the sensing pulse along the fiber. The bimodal structure is generally limited by the strength and length of heating or stress: generally, the greater the strength and length, the more obvious the bimodal structure. The frequency shift at the hot spot is usually obtained by bimodal fitting. In theory, the narrower the spectrum linewidth, the smaller the half-peak full width of the peak of the bimodal structure generated by the pre-pumped pulse in the temperature-sensing process, so the higher the precision of fitting at the hot spot position, the smaller the error, and the frequency resolution will be improved.

### 2.2. Curvature Measurement Principle of Multi-Core Fiber

The main idea of curvature measurement of a multi-core fiber is to use a fiber cluster curvature sensor made of multiple single-mode fibers combined in a fixed arrangement or a multi-core fiber to measure the strain information of the measured object in contact with it [6] and then to solve the measured strain information through the shape reconstruction algorithm to obtain the curvature information of the curved part.

**Figure 4:**
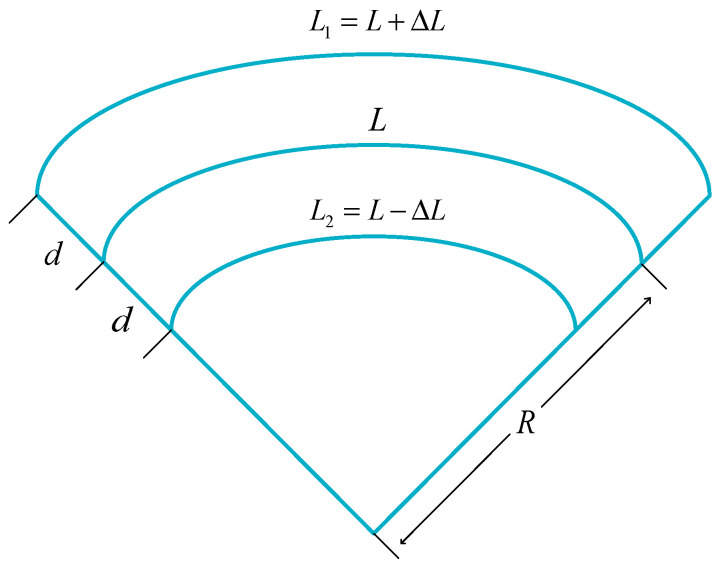
Simplified theoretical model of distributed shape sensor.

In this paper, an MCF [25] is adopted as the transmission medium, and the simplified theoretical model of the distributed curvature sensor is shown in Figure 4, where R is the bending radius of the fiber, L is the initial length of the sensor, d is the distance between the fiber core and the neutral axis of the sensor, and ΔL is the length of the fiber core being stretched and compressed.

If the stresses on the ***L*_1_** and ***L*_2_** sides are $\varepsilon_{1}$ and $\varepsilon_{2}$, then the stress variation on both sides should have the following relationship:

(1) $$\Delta \varepsilon_{1}=\frac{d}{R}=-\Delta \varepsilon_{2}$$

Taking the system based on a Brillouin scattered signal as an example, its Brillouin frequency shift (BFS) is affected by two variables, temperature and strain, where the temperature coefficient is $C_{T}$, strain coefficient is $C_{\varepsilon }$, and then its change can be expressed as follows [26]:

(2) $$\Delta v_{B}=C_{T}\Delta T+C_{\varepsilon }\Delta \varepsilon$$

Assuming that the multi-core fiber is subject to the same temperature at the same position, the Brillouin frequency shift of any two cores in the multi-core fiber can be subtracted to eliminate the effect of temperature on BFS, and Equation (3) can be obtained.

(3) $$\begin{array}{ll} \Delta v_{B2}-\Delta v_{B1} & =C_{T}\Delta T_{2}+C_{\varepsilon }\Delta \varepsilon_{2}-\left(C_{T}\Delta T_{1}+C_{\varepsilon }\Delta \varepsilon_{1}\right)\\ & =C_{T}\left(\Delta T_{2}-\Delta T_{1}\right)+C_{\varepsilon }\left(\Delta \varepsilon_{2}-\Delta \varepsilon_{1}\right)\\ & =-C_{\varepsilon }\frac{2d}{R}\\ & =-2C_{\kappa }\kappa \end{array}$$

where $C_{\kappa }=C_{\varepsilon }d$ is the curvature coefficient of the fiber and κ=1/R is the curvature of the fiber.

The Fleiner equation is the most commonly used 3D shape reconstruction algorithm, but when the curve has an inflection point, it is difficult to define the unique continuous Fleiner equation on the curve, resulting in low shape reconstruction accuracy. In this paper, a 3D parallel transmission frame is used. It consists of a tangent vector ***T*(*s*)**, a normal vector ***N*_1_(*s*)**, and another curvature component ***N*_2_(*s*)** along each point of the curve. ***T*(*s*)**, ***N*_1_(*s*)**, and ***N*_2_(*s*)** are composed of the following relations:

(4) $$\left[\begin{array}{c} T^{\prime }(s)\\ {N_{1}}^{\prime }(s)\\ {N_{2}}^{\prime }(s) \end{array}\right]=\left[\begin{array}{ccc} 0 & \kappa_{1}(s) & \kappa_{2}(s)\\ -\kappa_{1}(s) & 0 & 0\\ -\kappa_{2}(s) & 0 & 0 \end{array}\right]\left[\begin{array}{c} T(s)\\ N_{1}(s)\\ N_{2}(s) \end{array}\right]$$

where the direction of ***T’*(*s*)** is the bending direction of the curve. Then, the curvature κ(s) is defined as

(5) $$\kappa \left(s\right)=\left|T^{\prime }\left(s\right)\right|$$

It can be obtained according to Equations (4) and (5).

(6) $$\kappa (s)=\sqrt{{\left(\kappa_{1}(s)\right)}^{2}+{\left(\kappa_{2}(s)\right)}^{2}}$$

In the equation, $\kappa_{1}(s)$ and $\kappa_{2}(s)$ are the two curvature components of κ(s) in the directions of ***N*_1_(*s*)** and ***N_2_*(*s*)**, respectively, and can be obtained by interpolation fitting of $\kappa_{x}$ and $\kappa_{y}$.

## 3. Experimental System

**Figure 5:**
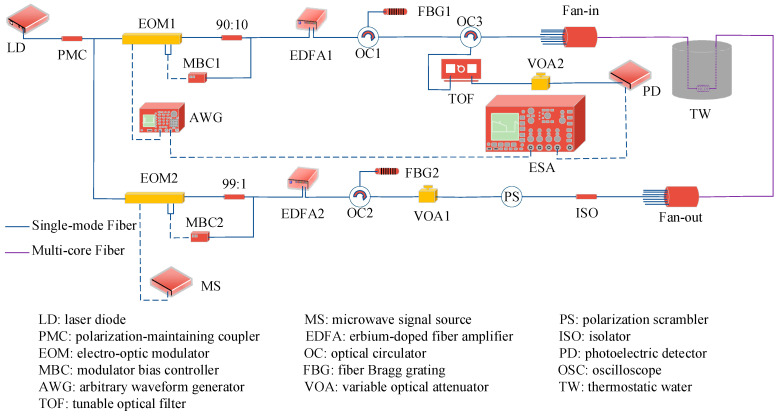
PPP-BOTDA curvature measurement system diagram.

The curvature measurement sensing system based on PPP-BOTDA was built, as shown in Figure 5. 

The system uses a narrow-band tunable Laser Diode (LD) as its light source, which has a spectral width of 100 kHz, an output center wavelength of 1550.056 nm, and an optical power of 13.5 dBm. A Polarization Maintaining Coupler (PMC) with a 50:50 splitting ratio divides the LD output into two branches: the upper branch for pumping light and the lower branch for detecting light. In this setup, pulsed pre-pumping light serves as the pumping light and continuous light serves as the detecting light. The upper branch directs the light into an Electro-Optical Modulator (EOM1), which is modulated by Arbitrary Waveform Generators (AWGs) to produce the pulsed pre-pumping light. EOM1 is locked at its valley point by a Modulator Bias Controller (MBC1). The intensity of the modulated pulsed pre-pumping light is then amplified by an Erbium-Doped Fiber Amplifier (EDFA1). Grating Filter 1 (FBG1) is used to filter out Amplified Spontaneous Emissions (ASEs). The pulsed pre-pumping light is sent through the circulator from port 1 and then enters the multi-core fiber via port 2 through a fan-in fan-out device. For the lower branch, a Microwave Generator (MG) drives EOM2 to generate a two-sideband signal with a suppressed carrier. EOM2 is also locked at its valley point by MBC2, and the continuous light is amplified by EDFA2. The amplified signal is filtered by FBG2 to remove ASE noise and anti-Stokes light. It then passes through a Polarization Scrambler (PS), which is followed by an adjustable attenuator. The light then goes through an Isolator (ISO) before entering the sensor fiber via the fan-in fan-out device, where it interacts with the pulsed pre-pumping light from the upper branch. The scattered light returns through the circulator from port 2 and then enters a Tunable Optical Filter (TOF) from port 3 to filter out the fundamental and anti-Stokes light of the probing light. After photoelectric conversion by a photodetector (PD), the lower band signal is fed into an Electric Spectrum Analyzer (ESA) set to “zero span” mode to collect the time-domain signal. 

## 4. Curvature Measurement and Result Analysis

### 4.1. Temperature Experiment of Single-Mode Fiber

#### 4.1.1. Spatial Resolution Measurement

To evaluate whether the PPP-BOTDA can achieve a spatial resolution finer than 1 m, we conducted a temperature verification experiment using a single-mode optical fiber. A 352 m long single-mode fiber was used, with the heating section located 8 m from the fiber’s front end and extending 1 m in length. The experiment was conducted at a room temperature of 20 °C, with the heating section immersed in a constant-temperature water bath set to 50 °C. Two sets of experiments were performed for comparison: one using standard 40 ns pulses and the other using the pre-pumped BOTDA with both 40 ns and 3 ns narrow pulses. 

**Figure 6:**
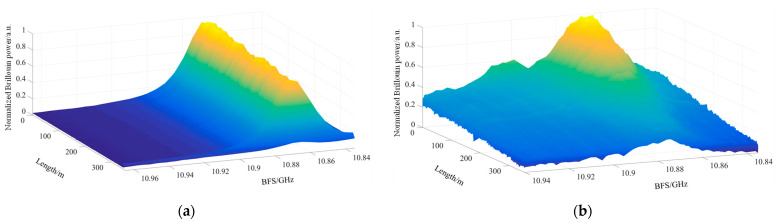
Normalized Brillouin gain spectrum: (**a**) normalized Brillouin gain spectrum for 40 ns pulsed BOTDA experiment; (**b**) normalized Brillouin gain spectra for 40 ns pre-pumped and 3 ns narrow pulse PPP-BOTDA experiments.

Figure 6 displays the normalized Brillouin gain spectra (BGS) for the two experimental setups. While the pre-pumping method theoretically offers improved spatial resolution, practical limitations arise due to the non-ideal rectangular shape of the pulse waveform. The modulation waveform is constrained by the rise and fall times of the AWG, which in this case were 800 ps. Consequently, a 3 ns narrow pulse was selected for the experiment.

Figure 6a presents the normalized Brillouin gain spectrum of the 40 ns pulse-modulated BOTDA system. The theoretical spatial resolution of this system is 4 m, and the heating section used in the experiment measures 1 m in length. The figure clearly shows that the system fails to detect the heating section. Figure 6b displays the normalized Brillouin gain spectrum for the PP-BOTDA system utilizing 40 ns pre-pumped pulses with 3 ns narrow-pulse modulation. The spectrum clearly illustrates changes in the Brillouin frequency shift within the heating section.

**Figure 7:**
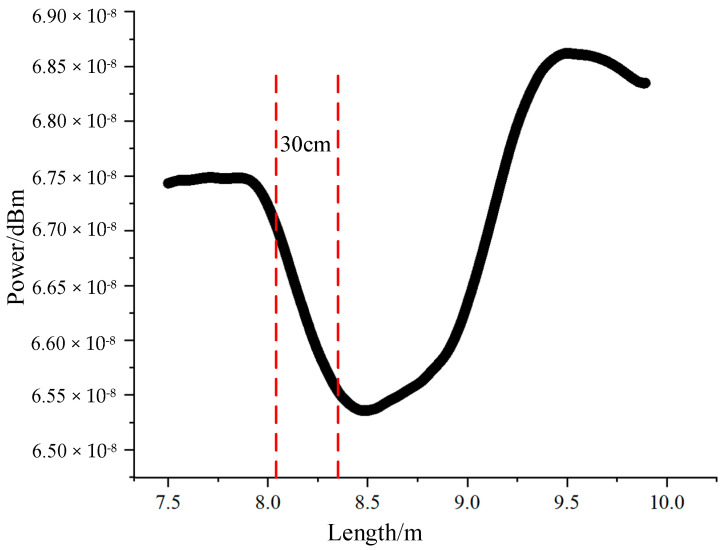
Time-domain curve of PPP-BOTDA.

Figure 7 depicts the time-domain curve of the PPP-BOTDA system. The curve indicates a spatial resolution of approximately 30 cm, which aligns with the theoretical spatial resolution of the 3 ns narrow pulse PPP-BOTDA system. 

#### 4.1.2. Bimodal Fitting 

**Figure 8:**
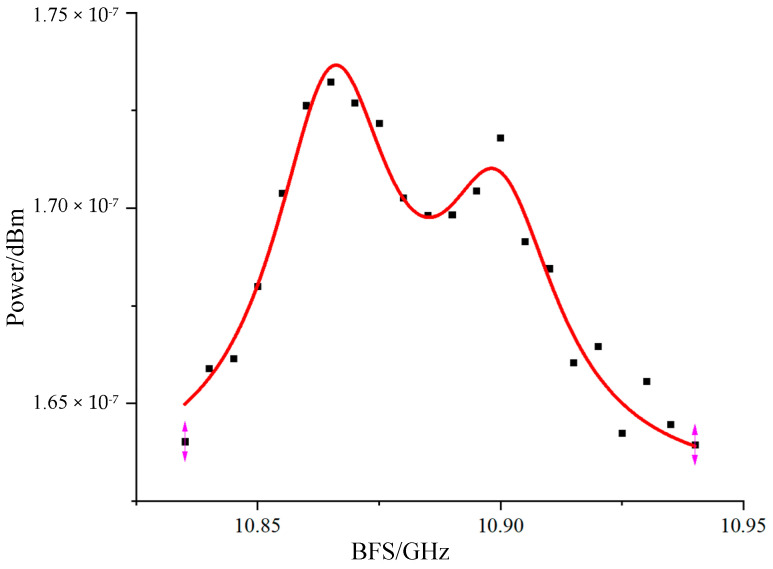
Bimodal Lorentz fitting curve for PPP--BOTDA system.

The BFS of the heating section in the optical fiber can be determined by extracting the Brillouin frequency shift from the PPP-BOTDA system and applying Lorentzian fitting to the time-domain curve, as shown in Figure 8. 

In this experiment, the BFS of the ordinary single-mode fiber at room temperature (20 °C) is approximately 10.868 GHz. When the heating section is placed in a water bath heated to 55 °C, the theoretical BFS for this section should be around 10.903 GHz. Figure 8 illustrates that the measured BFS of the heating section aligns well with the theoretical value for the single-mode fiber. After fitting, the Brillouin frequency shifts are found to be 10.865 GHz and 10.900 GHz, with corresponding Brillouin linewidths of 31.2 MHz and 29.6 MHz, respectively. The slight deviation between the fitted BFS values and the theoretical BFS of the single-mode fiber is attributed to errors introduced by the double-humped Lorentzian fitting. Additionally, it was observed that spectrum aliasing occurs when the two peaks are close to each other, especially at lower temperatures or smaller temperature differences, making it challenging to accurately distinguish between the BFS values of the two peaks. At higher temperatures, the larger double-peak separation allows for better differentiation and higher accuracy in Lorentzian fitting, resulting in smaller errors.

#### 4.1.3. Bilateral Tape Interference PPP-BOTDA System

The Brillouin frequency shift of the heating strain section is determined using a bimodal fitting method. In theory, a narrower spectrum linewidth results in a smaller half-peak width of the bimodal structure generated by the pre-pumped pulse during the sensing process. Consequently, this increases the precision of fitting at the hot spot, reduces errors, and enhances the frequency resolution. To address the issue of the double-peak phenomenon in the PPP-BOTDA, we employ the two-sideband interference method. This technique effectively narrows the peak linewidth and minimizes spectral overlap, thereby improving the accuracy of the measurement.

When the pump light of frequency is transmitted in the reverse direction relative to the probe light, which contains two frequency components ($\omega_{0}+\Delta \omega$ and $\omega_{0}-\Delta \omega$), the amplitude and phase of these components are modulated through the SBS effect, provided that the phase-matching conditions between the three laser beams are satisfied. In this process, the low-frequency component of the detected light is enhanced, while the high-frequency component is attenuated. Ignoring the effects of optical fiber loss and polarization, the output of the detected light after the acoustic–optical interaction can be described by Equation (7):

(7) $$\begin{array}{c} E_{\mathrm{out}}(t,\Delta \omega )=E_{1}\exp(j(\omega_{0}+\Delta \omega )t+j\varphi_{1})+E_{2}\exp(j(\omega_{0}+\Delta \omega )t+j\varphi_{1})\exp(H_{\mathrm{as}}(\omega_{0}+\Delta \omega ))\\ +E_{1}\exp(j(\omega_{0}-\Delta \omega )t+j\varphi_{2})+E_{2}\exp(j(\omega_{0}-\Delta \omega )t+j\varphi_{2})\exp(H_{s}(\omega_{0}+\Delta \omega )) \end{array}$$

In this context, $E_{1}$ and $E_{2}$ represent the complex amplitudes, while $H_{s}(\omega_{0}+\Delta \omega )$ and $H_{\mathrm{as}}(\omega_{0}+\Delta \omega )$ denote the complex Brillouin gain components at frequency ω, as indicated in Equation (8). Here, $G_{s}(\omega )$ and $G_{\mathrm{as}}(\omega )$ are the real parts of the Brillouin gain and loss at frequency ω, respectively, and $\varphi_{s}(\omega )$ and $\varphi_{\mathrm{as}}(\omega )$ are the imaginary components corresponding to the phases of the Stokes and anti-Stokes light. This relationship is further detailed in Equation (9) [21,27].

(8) $$\begin{array}{c} H_{s}(\omega )=G_{s}(\omega )+j\varphi_{s}(\omega )\\ H_{\mathrm{as}}(\omega )=G_{\mathrm{as}}(\omega )+j\varphi_{\mathrm{as}}(\omega ) \end{array}$$

(9) $$\begin{array}{c} G_{s}(\omega_{0}-\Delta \omega )=-G_{\mathrm{as}}(\omega_{0}+\Delta \omega )=\frac{g_{B}\Gamma^{2}}{4{\left(\Delta \omega -\Omega_{B}\right)}^{2}+\Gamma^{2}}\\ \varphi_{s}(\omega_{0}-\Delta \omega )=\varphi_{\mathrm{as}}(\omega_{0}+\Delta \omega )=\frac{{2g}_{B}(\Delta \omega -\Omega_{B})\Gamma }{4{\left(\Delta \omega -\Omega_{B}\right)}^{2}+\Gamma^{2}} \end{array}$$

In this context, $g_{B}$ represents the Brillouin gain coefficient, $\Omega_{B}$ denotes the Brillouin resonance frequency, and Γ indicates the intrinsic half-peak full width (FWHM) of the Brillouin spectrum. According to Equation (8), the two sidebands acquire opposite gains while maintaining the same phase during SBS modulation. As the two light beams interfere within the fiber, a narrow-bandwidth photodetector can be used to measure the double-sideband signal at the output end. The high-frequency components generated by the sideband beat frequency can be disregarded, and the coherent optical power detected by the photodetector $I_{\mathrm{out}}$ is represented by Equation (10).

(10) $$\begin{array}{l} I_{\mathrm{out}}(t,\Delta \omega )=E_{\mathrm{out}}(t,\Delta \omega )E_{\mathrm{out}}^{*}(t,\Delta \omega )\\ \;\;\;\;={\left|E_{1}\right|}^{2}+{\left|E_{2}\right|}^{2}+2{\left|E_{2}\right|}^{2}\cos(\varphi_{s}(\omega_{0}-\Delta \omega )) \end{array}$$

According to Equation (10), the detected optical power is influenced by the Brillouin phase, which in turn is affected by frequency detuning. By sweeping the frequency, the interference spectrum of the two sidebands can be obtained. The detector simultaneously captures information from both sidebands, involving two SBS processes: the interaction between the high-frequency sideband and the pump light and the interaction between the pump light and the low-frequency sideband.

**Figure 9:**
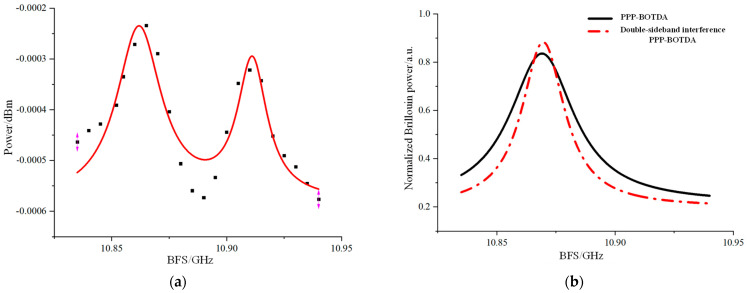
Experimental results of bilateral tape interference PPP-BOTDA: (**a**) bimodal Lorentz fitting curve for a two-sideband interference PPP-BOTDA system; (**b**) comparison of PPP-BOTDA and double-sideband interference PPP-BOTDA Lorentz fitting curves.

In the experiment, we replaced the FBG2 (central wavelength 1550.310 nm, bandwidth 0.370 nm) device with FBG3 (central wavelength 1550.125 nm, bandwidth 0.243 nm). This replacement allowed FBG3 to retain both sidebands of the suppressed carrier double-sideband signal generated by EOM2, while the TOF device was removed. As a result, the Brillouin signal entered the 1G bandwidth AC-coupled photodetector, which included both Stokes and anti-Stokes sidebands. The experimental results are shown in Figure 9. Figure 9a presents the Lorentzian fitting results for the double-sideband interference in the heating section, while Figure 9b compares the Lorentzian fitting results of the standard PP-BOTDA with those of the double-sideband-interference PP-BOTDA. Under the same room temperature and heating conditions, the double-sideband interference reveals more distinct peaks, narrower linewidths, and significantly reduced spectral overlap. After fitting, the Brillouin frequency shifts are found to be 10.863 GHz and 10.908 GHz, with 3 dB linewidths of 23.1 MHz and 16.1 MHz, respectively. This represents a significant improvement over the previous linewidths of 31.2 MHz and 29.6 MHz. The experimental results show a slight discrepancy between the fitted Brillouin frequency shifts and the theoretical values, attributed to the bimodal fitting using ORIGIN 2018 software. Although the fitting quality with the original data is somewhat lower than that of single-modal Brillouin Lorentz fitting, it still exceeds 80%, providing valid data.

### 4.2. Curvature Measurement of Multi-Core Fiber

**Figure 10:**
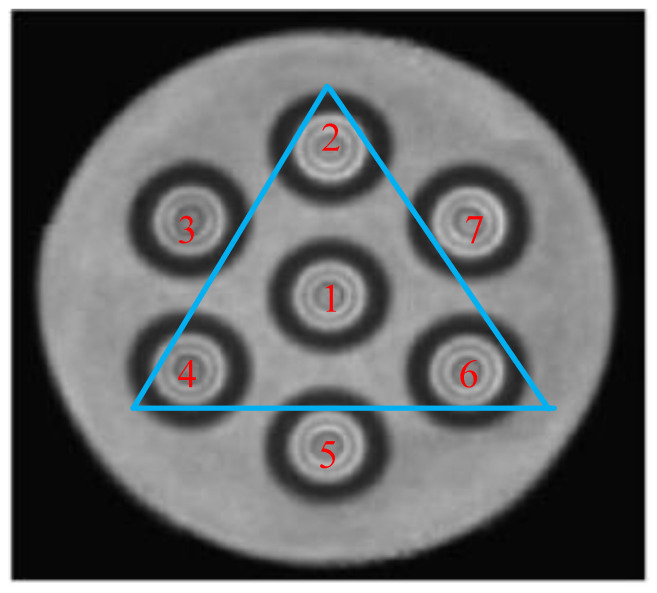
Internal structure diagram of seven-core optical fiber.

The feasibility of the PPP-BOTDA for bending measurements was demonstrated through single-mode temperature experiments. For these experiments, a homogeneous, low-crosstalk, multi-core fiber was used as the curvature sensor, with its internal structure illustrated in Figure 10. The fiber has a length of 290 m, a core diameter of 8 µm, a cladding diameter of 150 µm, and a protective layer diameter of 245 µm. In the sensor structure, one fiber core is positioned along the central axis, while the remaining six cores are symmetrically arranged around it, each 42 µm away from the central core and distributed at 60° intervals. Additionally, the seven pigtails of the multi-core fiber were labeled and separated using a fan-in fan-out coupler.

During the experiment, the inherent defects of the PPP-BOTDA and the characteristic that the middle core of the seven-core fiber is not affected by strain were taken into account [4]. When measuring the curvature of a seven-core fiber, the symmetric two cores will be stretched and compressed, so its BFS will be shifted to the long wavelength direction and the short wavelength direction accordingly. To more accurately measure the BFS after the seven cores are bent, the water bath is heated to the limit temperature, widening the frequency range between the two peaks of the PPP-BOTDA. We heated the bending section in a water bath set to 90 °C to ensure that the two peaks of the pre-pump signal were fully separated, allowing for the precise detection of small frequency shifts due to bending. By analyzing the experimental results of seven fiber cores, three asymmetric fiber cores with good peak fitting were selected for curvature reduction. We selected the offset cores 2, 4, and 6, as well as intermediate core 1, which are distributed 120° apart from each other. Additionally, intermediate core 1 was used for temperature compensation to mitigate the cross-sensitivity of temperature and strain in the eccentric fibers. It is worth noting that heating the water bath to 90 °C is an exaggerated experimental method, aiming to obtain the maximum frequency range between the two peaks as much as possible. It can be seen from the previous single-mode temperature experiment that it is feasible to select a suitable core for bending measurement at a certain temperature, and the method proposed in this paper can well solve the problem that temperature and bending strain are sensitive at the same time.

**Figure 11:**
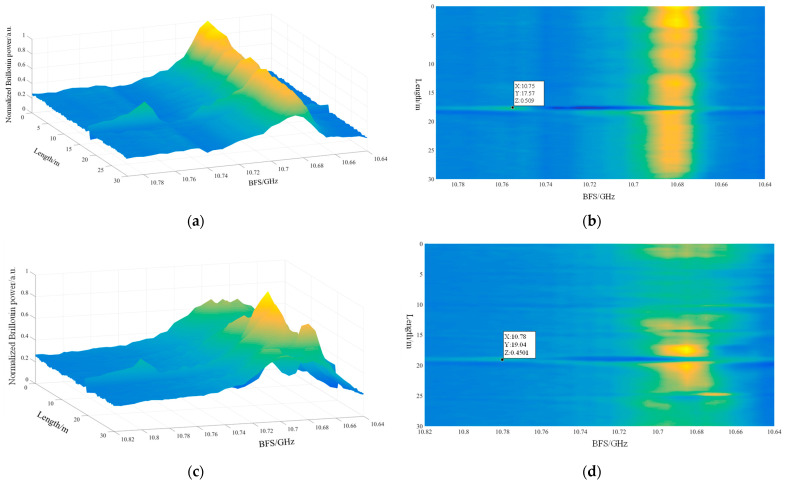
The experimental results of the middle core and off-core 6 in the 0–30 m bending section of the MCF: (**a**) the BGS three-dimensional and top view of the middle core; (**b**) the top view of the middle core; (**c**) the BGS three-dimensional view of core 6; (**d**) the top view of core 6.

We prepared a 40 cm bending strain section approximately 20 m from the front end of the seven-core fiber and wound it around a disk with a 10 cm radius, taking care to avoid torsion. BFS measurements were taken from the strain section of the four selected fiber cores. Due to the two-sided interference and double-peak nature of the PPP-BOTDA system, the first peak corresponds to the BFS at room temperature of the fiber core, while the second peak corresponds to the BFS at the hot spot location. In the data processing, we focused solely on the BFS variation of the second peak to measure the curvature accurately. Figure 11 shows the experimental results of the middle core and off-core 6 in the 0–30 m bending section of the MCF. Figure 11a,b show the BGS three-dimensional and top view of the middle core, respectively. It can be seen from the figure that the BFS of the fiber at room temperature (20 °C) is 10.683 GHz, because the middle core is not affected by bending. The BFS after heating is 10.752 GHz, and the 3D image is smooth. Figure 11c,d show the three-dimensional BGS diagram and top view of core 6, respectively. It can be seen from the figure that the BFS of the core deflection at room temperature is still 10.682 GHz, and the BFS after the simultaneous action of bending and strain is 10.781 GHz. At the same time, the signal strength of the core deflection is significantly lower than that of the intermediate core because the core deflection is affected by both bending and temperature. And because the rest of the MCF is wound around the optical fiber disc, the bending strain is different at each position, and the three-dimensional map will be much coarser.

During data processing, the BFS of each core was compared against the BFS of the middle core to eliminate the effects of axial strain and temperature on the bending measurements. This comparison yielded the BFS variations for the four cores. The BFS variations for cores 2, 4, and 6 were then substituted into Equation (2). For that experiment, we assumed a constant temperature, setting the parameters ΔT=0 and $C_{\varepsilon }$ to 0.0486 MHz/με [20]. It is worth noting that the strain coefficient of the MCF should be calibrated and measured before the experiment, but due to the limitation of experimental conditions, we directly quoted the data measured by Professor Zhiyong Zhao’s team in 2017 into the formula for calculation. This allowed us to determine the strain values for fiber cores 2, 4, and 6.

The strain values obtained for the three cores were input into the parallel transmission frame to determine the curvature components $\kappa_{1}$ and $\kappa_{2}$ of the fiber’s bending section. The curvature information was then calculated using these components in Equation (6). The results are presented in Table 1. From the experimental data, the calculated curvature of the bending section is 20.67 m^−1^, corresponding to a bending radius of approximately 4.84 cm. The measured bending radius is around 5 cm. The calculated radius is close to the actual measurement, with an error of about 3.2%. This discrepancy is attributed to two factors: first, the actual bending radius of the disk with a shallow groove is less than 5 cm; second, there is an inherent error in the bimodal Lorentz fitting of the PPP-BOTDA system measurement results.

In order to verify the effectiveness of the curvature measurement of the system, a curvature measurement with a diameter of 7 cm was carried out under the same measurement environment, and the results are shown in Table 2. Through calculation of the experimental data, the curvature of the bending section was 28.91 m^−1^, that is, the bending radius was about 3.45 cm, and the error was 1.45%.

## 5. Conclusions

In this paper, we present a PPP-BOTDA curvature measurement system. Due to constraints imposed by the pulse generator’s rise and fall times, we selected a combination of 40 ns long pulses and 3 ns narrow pulses for pre-pumping. Both the theoretical analysis and experimental results led to the following conclusions:

(1) Temperature experiments on the single-mode fiber confirmed that the PPP-BOTDA system can achieve a spatial resolution of 30 cm, significantly below 1 m under the specified conditions.
(2) The study of the bimodal Brillouin frequency shift in PPP-BOTDA revealed that the two-sided band interference method can enhance the accuracy of bimodal identification. This method effectively narrows the Lorentzian linewidth from 31.2 MHz and 29.6 MHz to 23.1 MHz and 16.0 MHz, thus reducing frequency aliasing in bimodal fitting.
(3) A homogenous, low-crosstalk seven-core fiber was used as a distributed curvature sensor. A 40 cm bending strain section, located approximately 20 m from the fiber’s front end, was wound around a disk with a radius of about 10 cm. Anti-torsion measures were applied during the winding process. The middle core was used for temperature compensation. By analyzing the Brillouin gain spectrum of the core and demodulating the stress variables, the curvature was calculated as 20.67 m^−1^; with a 3.2% error. The error is attributed to the shallow grooves in the disk and inaccuracies in bimodal fitting.

This paper verifies the feasibility of the proposed method in curvature measurement and provides a new idea for curvature measurement. At the same time, the proposed method is more suitable for solving the cross-sensitivity problem of temperature and strain. Of course, there are still many problems and limitations in this method, such as the inability to measure curvature alone at room temperature and certain errors in bimodal fitting. These are questions that need to be studied in the future.

## Figures and Tables

**Table 1:** Bending measurement results and calculation data with a bending radius of 5 cm.

Core Number	BFS/GHz	Change of BFS/GHz	Strain/με	Curvature/m^−1^
1	10.752	0	0	20.67
2	10.745	−0.00678	−139.506	
4	10.750	−0.00423	−87.037	
6	10.781	0.02931	691.564	

**Table 2:** Bending measurement results and calculation data with a bending radius of 3.5 cm.

Core Number	BFS/GHz	Change of BFS/GHz	Strain/με	Curvature/m^−1^
1	10.751	0	0	28.91
2	10.741	−0.01026	−211.11	
4	10.700	−0.00512	−105.35	
6	10.792	0.04086	840.74	

## Data Availability

All data reported in this paper are presented in the main text. Any other data will be provided on request.
